# Not of African Descent: Dental Modification among Indigenous Caribbean People from Canímar Abajo, Cuba

**DOI:** 10.1371/journal.pone.0153536

**Published:** 2016-04-12

**Authors:** Mirjana Roksandic, Kaitlynn Alarie, Roberto Rodríguez Suárez, Erwin Huebner, Ivan Roksandic

**Affiliations:** 1 Department of Anthropology, University of Winnipeg, 515 Portage Avenue, Winnipeg, Manitoba, R3B 2E9, Canada; 2 Department of Anthropology, University of Manitoba, 66 Chancellors Circle, Winnipeg, Manitoba, R3T 2N2, Canada; 3 Museo Antropológico Montane, Universidad de la Habana, Calle 25 No. 455 Vedado, Havana, 10400, Cuba; 4 Department of Biology, University of Manitoba, 66 Chancellors Circle, Winnipeg, Manitoba, R3T 2N2, Canada; University of Otago, NEW ZEALAND

## Abstract

Dental modifications in the Caribbean are considered to be an African practice introduced to the Caribbean archipelago by the influx of enslaved Africans during colonial times. Skeletal remains which exhibited dental modifications are by default considered to be Africans, African descendants, or post-contact indigenous people influenced by an African practice. Individual E-105 from the site of Canímar Abajo (Cuba), with a direct ^14^C AMS date of 990–800 cal BC, provides the first unequivocal evidence of dental modifications in the Antilles prior to contact with Europeans in AD 1492. Central incisors showing evidence of significant crown reduction (loss of crown volume regardless of its etiology) were examined macroscopically and with a scanning electron microscope (SEM) to determine if the observed alterations were due to deliberate modification or other (unintentional) factors considered: postmortem breakage, violent accidental breakage, non-dietary use of teeth, and wear caused by habitual or repeated actions. The pattern of crown reduction is consistent with deliberate dental modification of the type commonly encountered among African and African descendent communities in post-contact Caribbean archaeological assemblages. Six additional individuals show similar pattern of crown reduction of maxillary incisors with no analogous wear in corresponding mandibular dentition.

## Introduction

Dental modifications (DMs) in the Caribbean have been associated with individuals of African descent and, consequently, with the post-contact era [1–12]. The only exception is a skeleton recovered from the site of Chorro de Maita (Cuba), identified as a post-contact displaced Mesoamerican individual [13]. The latter shows a definite Mesoamerican type of dental filing, different in both style and technique from the “African-type” which predominantly involves crown reduction by chipping and filing of the upper anterior dentition [11, 14].

African practices of DM were first described in early accounts from European visitors to West Africa and later observed by ethnographers as summarized by Handler [6]. The most common forms of African DMs included chipping and filing of multiple incisors into points or ‘Vs’ and chipping and filing between upper central incisors resulting in an inverted ‘V’ shape [15–17].

**Fig 1:**
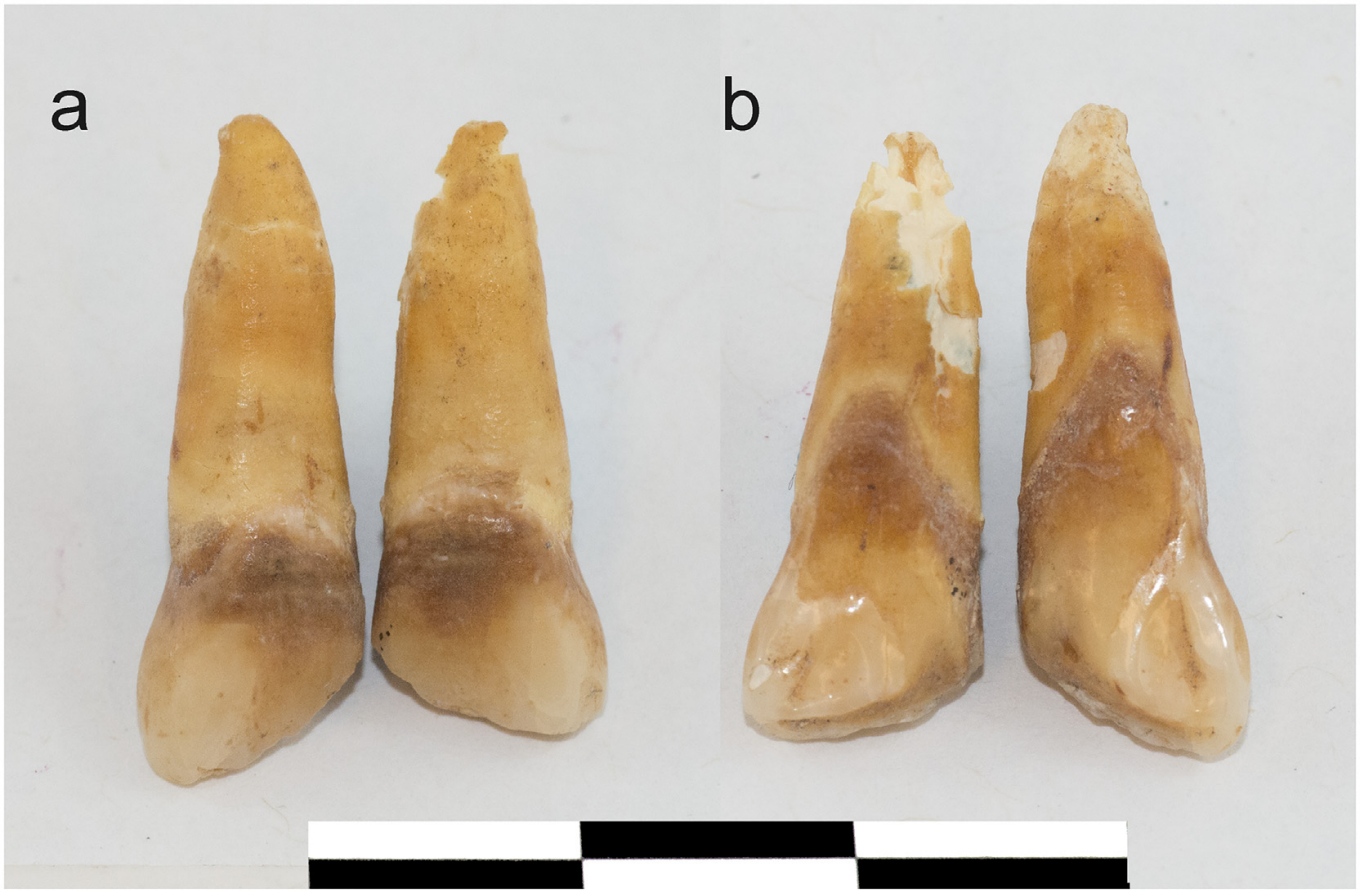
Modified central incisors of the individual E-105. Modified central incisors of the individual E-105 from the OC component, dated to 990–800 cal BC: a) labial view; b) lingual view (Photo by W. Hiebert, University of Winnipeg).

To date, no DMs in the Caribbean have been interpreted as evidence of a pre-contact practice, even when skeletal remains were recovered from indigenous cemeteries that predate contact [1]. Here we present the first case of the so-called “African type” DM observed in securely dated pre-contact individuals from Cuba, at the site of Canímar Abajo [18] predating the arrival of individuals from Africa to the Caribbean by almost 2.5 millennia [19]. Individual E-105, with a direct ^14^C AMS date of 990–800 cal BC [18] and an inverted “V” shaped crown reduction of central maxillary incisors (Fig 1), demonstrated that this type of DM was present in the Antilles prior to the arrival of enslaved African populations into the region.

## Materials and Methods

All necessary permits were obtained for the described study, which complied with all relevant regulations. Permit no PEA-2/14 to excavate and study the material from Canímar Abajo, Cuba, issued to Roberto Rodriguez Suarez on 24th of January 2014, by Comisión Nacional de Monumentos, Cuba. The permission includes transmission of material and data for study to other organizations with whom the team collaborates. Specimens are housed permanently and publicly at the University of Havana, Museo Montané; they are available for examination upon request to Roberto Rodríguez Suárez. Dental specimens presented in the paper are Canímar Abajo specimens: E-105 (4) LI1, RI1; E-125 LI1, RI1; E-19 LI1; E-92 (38) LI1, RI1; E-10 LI1, LI2, RI1, RI2; E-71 (10) LI1, LI2, RI1, RI2; E-23 (16) LI1, LI2, RI1, RI2.

All adult skeletons recovered from Canímar Abajo cemetery with preserved dentition (88 individuals) were examined macroscopically for evidence of dental modifications: 22 from the older cemetery (OC), 59 from the younger cemetery (YC), and seven individuals from which the stratigraphic context was ambiguous. Of these, only 46 individuals were sufficiently preserved (i.e. had both maxillary and mandibular anterior dentition) to be included in the study, as lack of corresponding wear is an important criterion in differential diagnosis.

For all individuals which exhibited altered incisors, the pattern of crown reduction (loss of crown volume regardless of etiology) was examined against several possible explanations [20]: postmortem breakage; accidental violent breakage; use wear (modification due to repeated non-masticatory activity); and deliberate dental modification. A number of isolated upper central incisors with apparent crown reduction were observed; they were not included in the final discussion as they could not be associated with the rest of their respective dentition.

Dentition of seven individuals with possible deliberate modification of the maxillary incisors was examined with a hand-held (Dino-lite) digital microscope under 20x magnification in the Anthropology Department at the University of Havana. Four specimens were available for examination at the University of Winnipeg using a higher resolution digital microscope Keyance (VHX-5000 series) at 20x magnification; central incisors of the individual E-105 were subject to scanning electron microscope (SEM) at x80 and x100 magnifications, as the altered surface was minimally worn. Both central incisors were examined with a Hitachi TM-1000 tabletop scanning electron microscope (SEM), housed in the University of Manitoba Department of Biological Sciences Microscopy Imaging Facility. This microscope obtains images using a backscattered electron detector, so that uncoated samples can be viewed. The teeth were mounted on aluminum SEM stubs with ultra-smooth carbon adhesive tabs on the surface to which the teeth adhered. Images were taken of both the lingual and labial surfaces horizontally. To obtain images of the occlusal edge, the teeth were oriented at an angle with the occlusal edge raised more vertically on the stud. The tooth was held up by a supporting pad of SEM carbon adhesive tape under the end of the tooth.

### Archaeological Context

**Fig 2:**
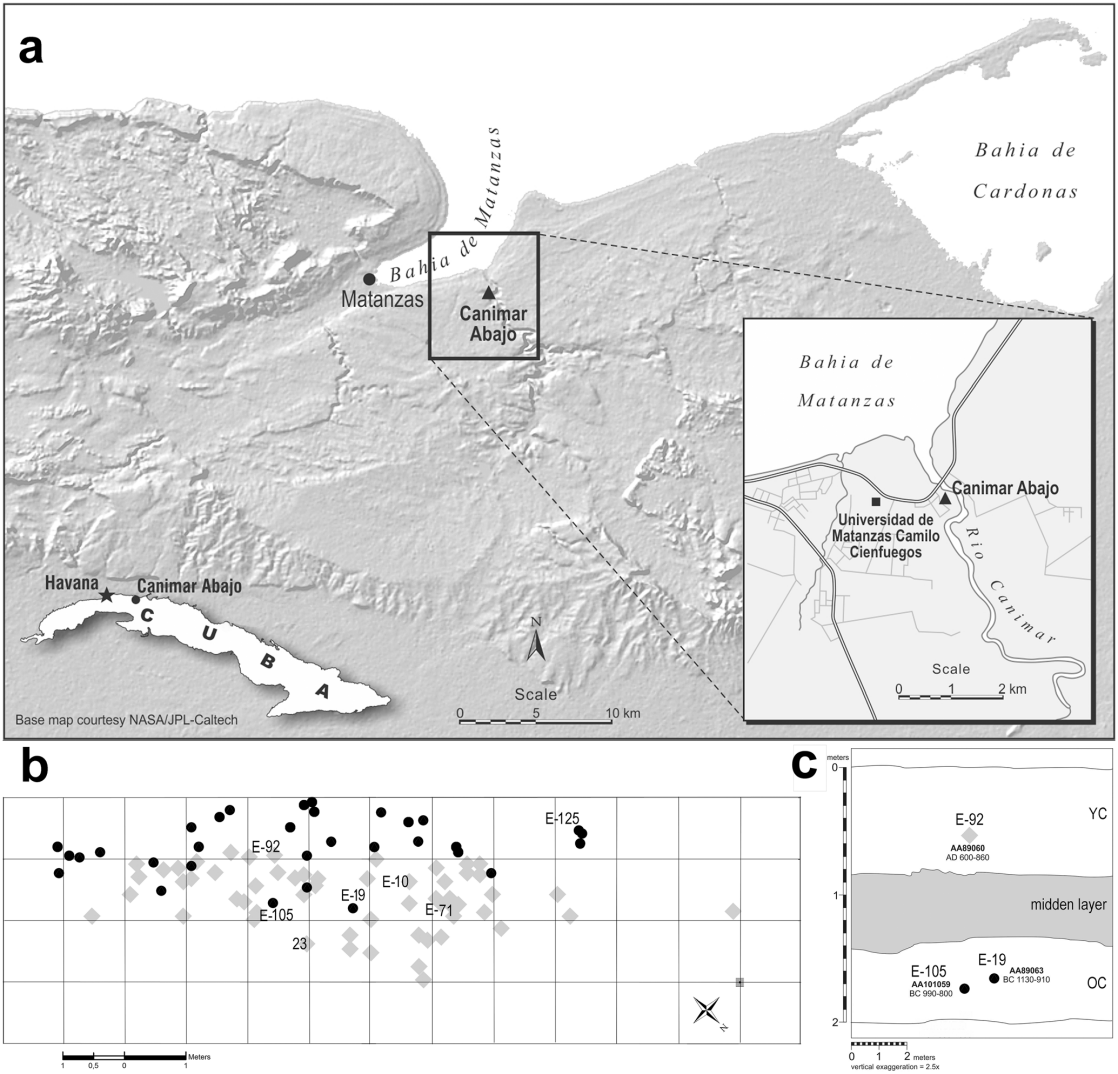
Location of the site of Canímar Abajo and the distribution of burials. The site of Canímar Abajo: a) location of the site at the Bay of Matanzas and on the map of Cuba (outlined in the lower left corner). Reprinted from [18] under a CC BY licence with permission from the University of Arizona, original copyright (2015); and uses the open source satellite map (http://www.jpl.nasa.gov/imagepolicy/); b) horizontal distribution of burials on the excavation grid of the site and the location of individuals discussed in the text: black dots represent Older Cemetery (OC) burials; gray rhombs represent Younger Cemetery (YC) burials; c) schematic representation of the profile of Canímar Abajo with dated burials discussed in the text. Black dots indicate OC burials; gray rhombs indicate YC burials. ^14^C laboratory numbers are given with their corresponding dates.

Individual E-105 was recovered in 2010 from the site of Canímar Abajo located near Matanzas city (23° 2' 15.5" N; 81° 29' 49.1" E) in the Matanzas province of Cuba (Fig 2a). Canímar Abajo is a complex shell-matrix site with two superimposed burial episodes separated by a midden layer [18]. The site is located on an ancient beach on the western bank of the Canímar River, near to where the river flows into the Bay of Matanzas, forming a resource-rich estuary [18]. Systematic excavations over 36 m^2^ (Fig 2b) yielded a minimum number of 213 individuals in 50 burials of the older cemetery (OC) and 92 burials of the younger cemetery (YC), as well as some isolated bones recovered from the midden layer (Fig 2c). The older of the two cemetery components was dated by six AMS ^14^C dates to between 1380–800 cal BC (2 sigma), while the younger was dated to cal AD 360–950 (2 sigma) by five AMS ^14^C dates obtained directly from human skeletal remains [18], all clearly predating the contact with European colonizers and the arrival of enslaved Africans into the Caribbean.

Individual E-105 was buried in the older cemetery (OC), consistent with the AMS ^14^C date obtained directly from the skeletal remains (AA101059) and calibrated to 990–800 BC (Fig 2c) [18]. The body was buried in ventral decubitus (face down), with both legs slightly flexed at the knees and positioned to the side of the axial elements of the body. The splaying of the rib cage indicates a relatively large burial space with no constriction [21]. There was no indication of post-depositional manipulation or disturbance of the body. The burial was covered by a layer of hardened matrix composed of shells, ash and dirt.

The dentition of E-105 is almost complete (S1–S3 Figs). The preserved mandibular alveoli indicate the third molars were fully erupted (S2 Fig). Individual E-105 was determined to be of young adult age [22] based on an incomplete fusion of the iliac crest and other secondary centers of ossification of the pelvis, clearly visible epiphyseal lines on the surviving long bones, and the full eruption of the third molars in conjunction with observed moderate degree of dental wear. Female sex was assigned on the basis of a wide sciatic notch, cranial morphology, and generally gracile skeletal remains [23].

Six more individuals with altered maxillary incisors were identified: E-19 (with a ^14^C AMS date of 1130–910 cal BC) and E-125 were recovered from the OC level; while four individuals: E-92 (with a ^14^C AMS date of cal AD 600–860), E-71, E-10 and E-23 were excavated from the YC level [18]. They present similar pattern of crown reduction involving the upper central (and in three cases also lateral) incisors in an inverted “V” pattern (Table 1), although the case for deliberate modification is more difficult to ascertain in these cases because of subsequent dental wear, which prevented a more thorough examination like the one offered for the E-105. The skeleton of a fully adult woman (E-125) deposited in dorsal decubitus (laying on the back) was heavily disturbed above the knees, possibly by a diachronous placement of a child burial on her pelvic region. The E-19 is a very poorly preserved individual buried in dorsal decubitus, in a shallow oval burial pit with a layer of rocks and charcoal above the burial. In the younger cemetery, individual E-92 (S4 Fig) was buried in ventral decubitus with the skull on its left side and legs extended. This position is reminiscent of the position of E-105, although the latter is buried face down and with slightly flexed legs. The Individual E-71 (S5 Fig) is represented only by a fragmented gracile skull within a group burial. Individuals E-23 (a young adult female) and and E-10 (a fully adult female) are positioned in relatively tight pits on right side, or in dorsal decubitus respectively, with knees drawn towards the thorax.

**Table 1:** Individuals with identified deliberate dental reduction of central and lateral incisors.

Individual	Layer	Teeth	Sex	Age	Calibrated ^14^C date
E-105 (4)	OC	LI^1^, RI^1^	Female	YA	990–800 cal BC
E-125 (2)	OC	LI^1^, RI^1^	Female	FA	
E-19	OC	LI^1^	Female	YA	1130–910 cal BC
E-92 (38)	YC	LI^1^, RI^1^	Likely Female	FA	cal AD 600–860
E-10	YC	LI^1^, LI^2^, RI^1,^ RI^2^	Female	FA	
E-71 (10)	YC	LI^1^, LI^2^, RI^1,^ RI^2^	Likely Female	FA	
E-23 (16)	YC	LI^1^, LI^2^, RI^1,^ RI^2^	Female	YA	

Individuals with identified deliberate dental reduction of central and lateral incisors. YA = Young Adult; FA = Full Adult (for definition of age stages see 22); L = left; R = right; I^1^ = central upper incisor; I^2^ = lateral upper incisor; OC = Older Cemetery; YC = Younger Cemetery.

## Results

A total of seven individuals from Canímar Abajo exhibited an inverted “V” pattern of reduction of maxillary incisors (Table 1). Possible cases of similar incisor reduction were observed in another seven isolated teeth. These were not included in the analysis as one of the criteria for distinguishing dental modifications from a repeated non-masticatory use is the lack of corresponding wear on the mandibular dentition. Among the seven individuals, E-105 showed the least alteration of the modified surface by subsequent dental wear, and thus allowed a more in-depth analysis.

### Description and differential diagnosis

Macroscopic, as well as low resolution and SEM microscopy are used to describe the altered incisal edge of maxillary central incisors in the individual E-105. Different attributes observed are discussed in comparison with evidence of postmortem trauma, violent trauma, non-intentional chipping produced by masticatory and dietary activities, and to those produced by habitual or repeated non-masticatory use of teeth.

Macroscopic observations: Both upper central incisors of individual E-105 were modified. The mesial aspects of the crown have been reduced on a 45 degree angle in a mesial to distal direction, exposing dentin across the modified incisal edge. The negative space formed between the modified incisors resembles an inverted “V” shape. There is no analogous wear on the lower incisors (S3 Fig), and no other teeth in the dental arcade showed signs of alteration or significant wear. While occlusal variation can sometimes produce anomalous wear patterns, the moderately rotated lower left canine in this individual resulted in no major issues for occlusion; as such, this can be excluded as an explanation. The presence of dental abrasion on the modified surface along the incisal edge excludes the postmortem breakage as an explanation. While the symmetrical pattern of crown reduction argues against a violent accidental breakage, it cannot be fully excluded on the basis of macroscopic observations alone, and will be discussed in conjunction with microscopic evidence.

The changes to the incisal edge do not conform to any of the degrees of chipping proposed by Bonfiglioli et al [24]. In contrast to the definition offered by Bonfiglioli et al. ([24]:449) as “an ante mortem irregular crack involving enamel or enamel and dentine, situated on the buccal, lingual or interproximal edge or crest of the tooth,” the incisor crowns of E-105 are reduced in a regular and symmetrical fashion across the two adjacent teeth (Fig 1).

Extramasticatory notching is unintentional, produced by repeated use of teeth to process or hold non-food items [25]. The angle of the inverted “V” shaped modification exhibited by E-105 (Fig 1 and S2 Fig) is superficially similar to the notch pattern observed by Molnar (see [25]: 683, Fig 1A); this is not surprising as both are symmetrical alterations of adjoining teeth. Bonfiglioli et al. [24] define notching as “an indentation involving the tooth’s incisal/occlusal edge.” ([24]:449) and stipulate that the depression should have a perpendicular or transverse orientation to the mesiodistal axis of the tooth. The case reported by Molnar [25] is consistent with Bonfiglioli’s [24] definition discussed above; however, it is labio-lingually oblique in the case of E-105. More importantly, repeated /habitual holding of an object between the anterior teeth would have left traces on both maxillary and the mandibular incisors, and therefore, a corresponding wear would be expected in the mandibular dentition. A very small object—that could have been held between incisors (and thus not involve mandibular dentition) would leave interstitial wear and present itself as a groove and not a notch [24].

Macroscopically observed characteristics of E-105 incisors do not conform to repeated non-masticatory usage of the teeth: chipping is regular and symmetrical as opposed to irregular and haphazard; the notching is much larger than could be produced by a repeated holding of a very small object; a larger object would produce corresponding wear on mandibular dentition. The location, symmetry, and most importantly, the lack of corresponding wear on the mandibular dentition, indicate that this was not a result of daily use-wear, habitual behaviors such as pipe smoking, or of using the teeth as tools [4, 26].

**Fig 3:**
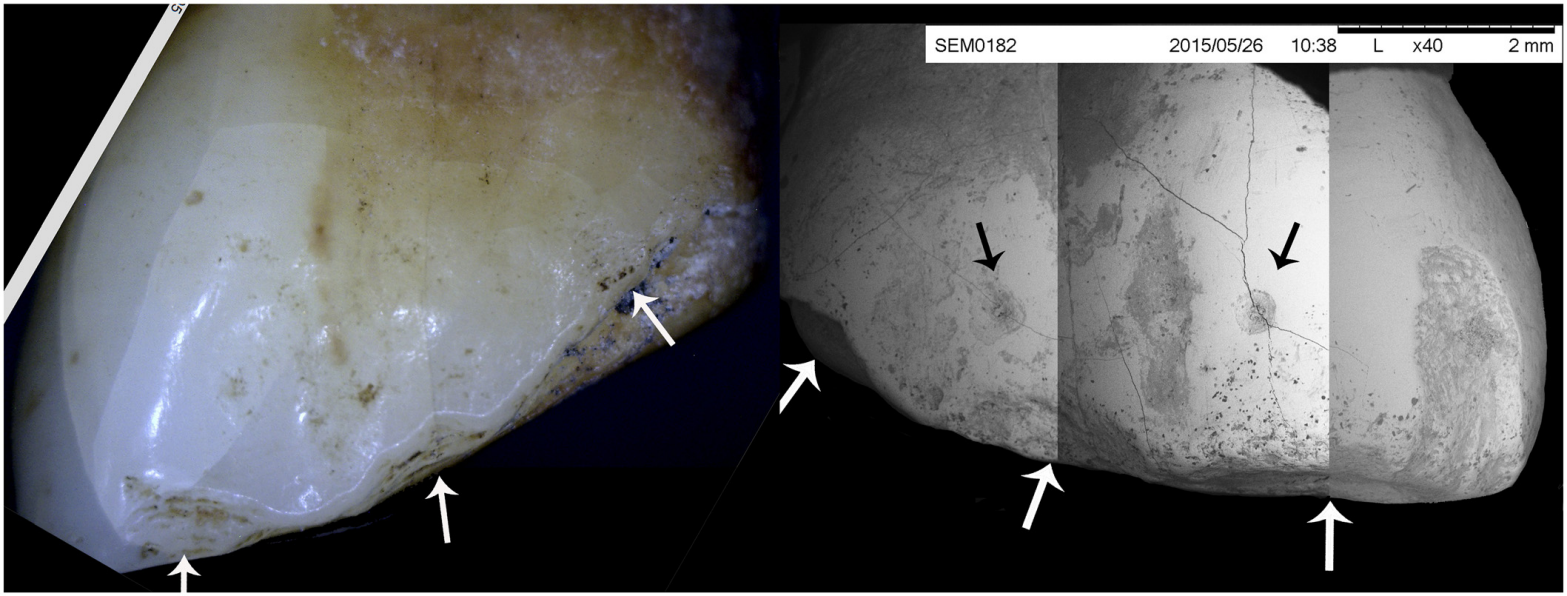
Labial surface of the maxillary central incisors of E-105. Labial surface of the maxillary central incisors of E-105a) under 20x magnification captured by digital microscope; b) composite image captured by an SEM microscope at 80x magnification. White arrows indicate the site of conchoidal fractures; black arrows indicate the pits on the enamel above the conchoidal fractures.

Microscopic observations: When the modified edge of E-105 was viewed under digital (20X) and SEM (40X) magnification (Fig 3 and S6 Fig), we observed evidence of chipping along the labial face of the modified edge, as well as hairline cracks on the labial crown enamel emanating from the modified edge towards the cemento-enamel junction (CEJ). Hairline cracks are often associated with high loading event, such as chipping [27]; however, they cannot be distinguished from similar lines produced through masticatory action. The modified surface remained sharp (or slightly jagged), indicating minimal subsequent wear of the chipped edge (Fig 1a and S6 Fig). No chipping can be observed on the lingual surface (Fig 1b and S7 Fig), which shows substantial abrasion of the enamel from mesio-incisal towards lateral portion of the CEJ, covering the lower two thirds of the tooth. The abrasion covers only the mesial third of the modified incisal edge, and does not extend onto the labial surface. Chipping seems to be subsequent to abrasion. There is no lingual abrasion observed on other maxillary and mandibular teeth.

Chipping of the incisal edge shows localized depressions with concentric lines (Fig 3) which are not observed in dentition chipped by processing hard foods [28, 29]. These depressions are reminiscent of conchoidal fractures, which are the hallmark of intentional percussive modification in both lithics and bone [30]. Three such depressions on each tooth can be observed both under low (Fig 3a) and high (Fig 3b) magnification. The most mesial of the depressions are continuous across the two teeth and might have been produced by a single strike: thus a total of five percussion events would be needed to produce the resulting pattern. Such a regular and repeated pattern, excludes the possibility that the reduction is a result of accidental (or violent) trauma.

In addition, when viewed using SEM microscopy (Fig 3b and S6 Fig) small and regular circular pits of equal size can be observed above the conchoidal depressions. Given the radiating fracture lines emanating from these pits, as well as concentric lines within them, these pits do not represent localized enamel hypoplastic growth disturbances. Their shape and characteristics are more consistent with a pattern of damage caused by pressure exerted by a hard pointed object. While they could be unrelated to the crown reduction, this is highly unlikely, because of their similar size, and location at regular intervals, directly above the conchoidal fractures (Fig 3b) where the modification has occurred. While pits caused by pressure can be detected on the SEM images of the occlusal surface from biting on hard objects [29], there is no reported record of these pits on the labial surface, which is normally not involved in mastication.

**Fig 4:**
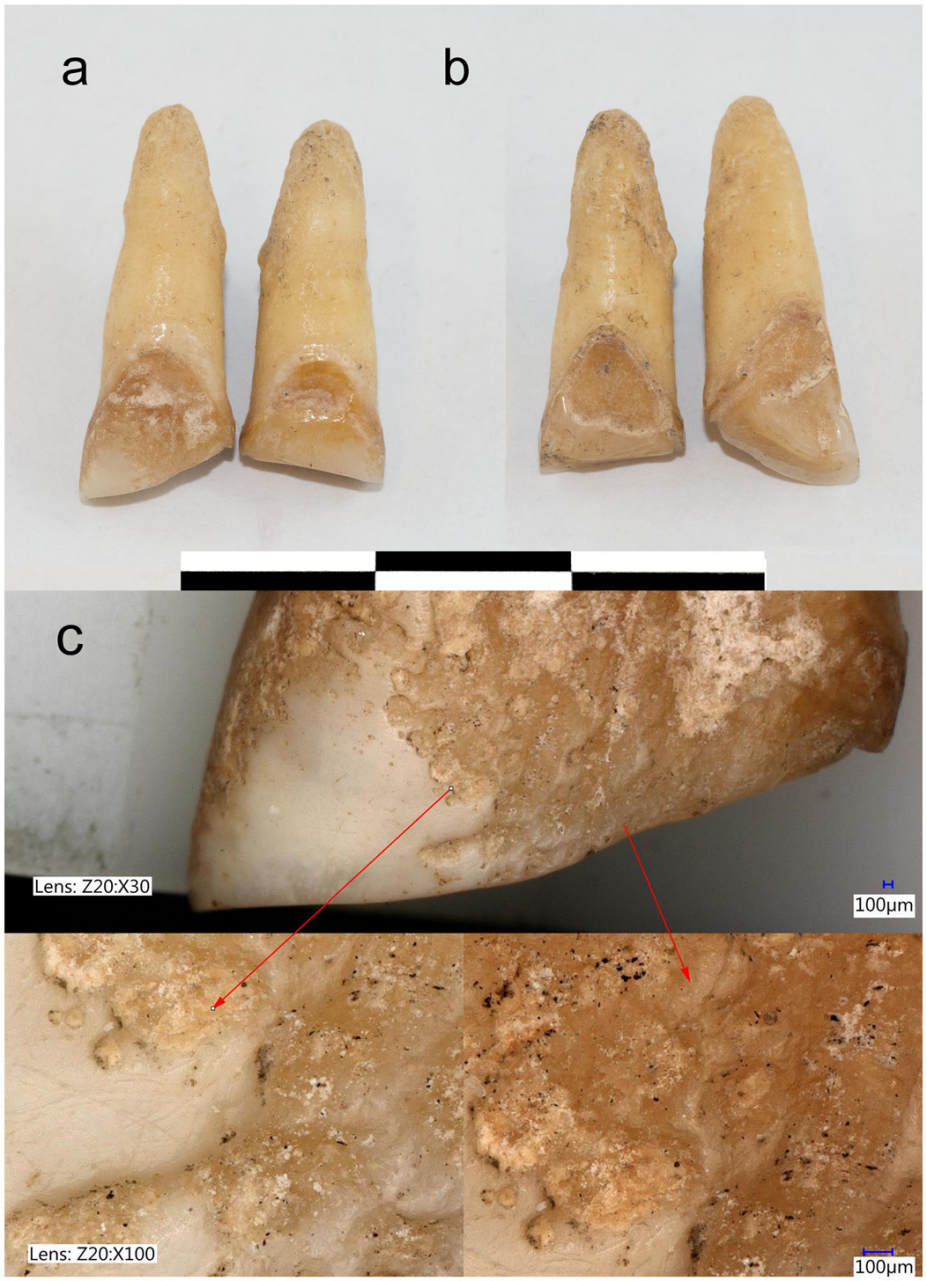
Modified maxillary central incisors of the individual E-92. Modified maxillary central incisors of the individual E-92 from the YC component dated to cal AD 600–800: a) labial view; b) lingual view (Photo by W. Hiebert, University of Winnipeg); c) taphonomic changes observed on the labial surface of the modified right maxillary central incisors of the individual E-92 from the YC component dated to cal AD 600–800 (images captured by Keyance digital microscope (VHX-5000 series) at 30x and 100x magnification).

Macroscopic examination of other six individuals represented in Table 1, shows a similar shape of teeth modified to resemble an inverted “V” involving only the upper central incisors (3 cases) or both central and lateral upper incisors (3 cases). In most of these samples dental wear is too advanced to present all of the characteristics observed in E-105. Faint traces of conchoidal fractures are still visible in individuals E-125 and E-19 (S8 Fig), while the surface of E-92 is too eroded (taphonomically) to ascertain their presence (Fig 4 and S8 Fig).

## Discussion

Crown reduction of central maxillary incisors of individual E-105 into an inverted “V” shape is most consistent with deliberate dental modification. This is demonstrated primarily by symmetrical conchoidal fractures on the labial surface spaced at regular intervals. These fractures are consistent with chipping: post-mortem trauma and violent trauma can be excluded on the basis of the shape of chipped areas, number of chipped areas, and the regular interval at which they were produced. Furthermore, the nature of chipping and grooving is not consistent with repeated or habitual non-masticatory use of teeth, since such behavior would have left evidence on the corresponding mandibular teeth. While the state of preservation and dental wear did not allow us to present the same in-depth analysis for each of the additional occurrences of incisive reduction in the sample, those elements that could be observed are consistent with the conclusion reached for the individual E-105. In particular, all of them present the same form of crown reduction, coupled with a complete lack of involvement of the mandibular dentition. While the most parsimonious explanation is that they represent deliberate DMs sanctioned by cultural practice, it cannot be ascertained.

The above elements are consistent with DMs using the chipping and filing technique [11]. No labiolingual striations on the modified edge could be observed under magnification, which has been proposed by Havill and colleagues [31] as evidence of the use of a sharp abrading tool and indicative of a filing episode. Such labiolingual striations, however, would have been obliterated fairly quickly in the course of regular dental abrasion associated with daily use of the dentition [32]. Accordingly, they are not considered necessary to demonstrate DMs. Given the chipped aspect of the labial crown surface, the modification of the teeth would have happened relatively close to the death of the individual E-105, although the abrasion of the occlusal edge of the teeth indicates that some time elapsed between the alterations and the time of death. Given the number of factors that remain unknown about the process, it is not possible to estimate the exact timing of the event.

In the individual E-105, the lingual surface of the central maxillary incisors shows substantial abrasion of the enamel involving the lower third of the crown (Fig 2b and S7a Fig). It proceeds from the mesial tip of the occlusal surface and extends towards the CEJ, at which level it covers the whole lingual surface. None of the other individuals with modified incisors show this pattern of lingual abrasion: the labial surface of the central incisors of individual E-92 is taphonomically altered (S8 Fig), while the other modified teeth are too worn to allow observation. Lingual surface attrition of the maxillary anterior teeth (LSAMAT)—often involving canines—purportedly results from processing fibrous, abrasive foods with high carbohydrate content (i.e. manioc, maize) [33,34]. Where it was recorded, it affected both sexes and a high proportion of the population: 82% of adults in Brazilian [33]; 57% in Panamanian [34]; 64% in Mayan [35]; and 45% in West African [36] skeletal populations. It remains unclear if there is any relationship between lingual wear on the central incisors and the DM in the individual E-105. It is however, evident that the observed crown reduction is not the result of the abrasion, and that the DM event occurred after the abrasion of the lingual surface has already taken place.

The pattern of chipping and the direction of conchoidal fractures on the labial surface, seem to indicate that the force was applied from the lingual direction, with a pointed object (or objects) holding the tooth in place during impact, resulting in localized pits on the labial surface. As there are no detailed descriptions that include SEM analyses of the chipping and filing techniques to which we could compare our results, our description is based solely on the location and the force of the percussive events. Further research is needed to ascertain the exact process by which this was accomplished.

Of the 46 individuals that met the criteria for inclusion in the study (18 from the OC and 28 from the YC), 20 individuals (44%) were identified as either male (based on pelvic traits) or likely male (based on cranial traits); 13 individuals (28%) were identified as female or likely female; while sex could not be assigned to 13 individuals (28%) due to the fragmentary nature of their pelvic and cranial remains. The overrepresentation of males is most likely due to a preservation bias. DMs were observed in seven individuals, representing 15% of the total sample, with a relatively equal distribution between the OC (17%) and the YC (14%). Interestingly, all dental modifications were observed on individuals identified as women. DMs were recorded in 54% of the total sample of identified adult women: three out of four (75%) from the OC, and four out of nine (44%) from the YC. Given this small sample size, the differences in the frequency of dental modifications among women between the OC and the YC cannot be discussed; however, it seems likely that deliberate DMs were a well-established practice reserved to adult women at Canímar Abajo.

DMs at Canímar Abajo span both cemetery components, which lasted between approximately 1400 BC and AD 950 with an apparent burial hiatus from 800 BC to AD 360 [18]. Long persistence of this type of modification could indicate that the same population used both cemetery components. This notion is further supported by the consistency of subsistence strategies employed in both the OC and YC at Canímar Abajo, as well the marked differences in subsistence strategies observed between Canímar Abajo and other contemporaneous Cuban sites [37]. Further research into the cultural meaning of body modifications in the region—for both past and present populations—is needed before we can discuss the motivation behind the DM practice at the site of Canímar Abajo. Analysis of dental morphology and the aDNA, which are currently underway, will provide more definite answers to the questions of continuity between the two components and their biological identity. While the ancestry of Canímar Abajo individuals cannot be ascertained, it is clear from associated ^14^C dates that they are indigenous Caribbean people and not enslaved Africans.

African origin of the practice of dental chipping and filing can be ascertained in a number of individuals from archaeological sites in the circum-Caribbean [7–11]. In a recent paper, Schroeder and colleagues [12] obtained a whole genome sequence for three individuals from Saint Martin, tracing their origins to distinct source populations from present day Cameroon, Nigeria, and Ghana. Slave trade records cited the presence of various DMs among enslaved Africans and their immediate descendants, with filing or chipping of the mesial and/or distal sides of the anterior dentition—affecting predominantly the upper incisors—being the most commonly practiced technique [11].

However, in a number of cases, African origin is assumed despite contextual information pointing to the indigenous origin of the examined individuals. This is illustrated by the case of two skeletons excavated from a shell midden in the U.S. Virgin islands for which Buxton and colleagues [38] proposed pre-contact indigenous affiliation based on the archaeological context and associated artifacts. T. D. Stewart [1] criticized Buxton and colleagues for assuming that the skeletons were indigenous only because they were found within an Amerindian shell-midden. In Stewart’s view, the key feature demonstrating that these skeletons had to be of African origin was the presence of DMs similar to those he observed in skulls from Barbados. The skulls from Barbados were, on the basis of these very DMs, identified by Stewart as enslaved Africans, despite being excavated from a midden site previously identified as “Arawak” ([1]:49).

Given T.D. Stewart’s influence as a prominent physical anthropologist and the lack of unequivocal evidence for the indigenous practice of DMs in the Caribbean, all individuals exhibiting DMs characterized by chipping and filing of the incisors and canines into points were considered to have been of African origin. Individuals from the Caribbean exhibiting this type of DMs were interpreted as first generation Africans [2–4, 6, 10] their immediate descendants [9, 11], or in some cases, indigenous people who adopted an African practice [3, 39]. The direct dating of the individual E-105 to the beginning of the first millennium BC demonstrates that this practice was present in the Caribbean before the initial contact and subsequent slave trade. As a result, African origin cannot be ascribed to excavated individuals displaying DM, unless it is supported by additional data.

There is no reported evidence of the presence of this type of DM among indigenous populations inhabiting the adjacent continental regions of the Caribbean basin in the pre-contact period. DMs involving crown reduction of central incisors are currently practiced among Palikur women in the state of Amapa (Brazil) and in French Guiana (van den Bel, personal communication July 28, 2015), and we are currently planning an ethnographic study with the community members to understand the origins, motivation and meaning behind the practice.

There are reports of “African-style dental mutilation” in South America in recent times. In 1918, William Farabee [40], while describing practices of Arawak people from Guyana, noted that “men filed the front teeth to a point.” A similar practice was observed among living indigenous groups in Panama, and interpreted as a consequence of the contact between indigenous groups in the region and runaway African slaves [39]. In support of this argument the author stated that “(since)…none is known (among indigenous people) from the days of the Conquest with its wealth of literary sources, tooth-filing must be ascribed to the Negroes” ([39]:328). Given the low social status that colonial societies ascribed to runaway slaves, it is unlikely that indigenous groups would have adopted their social customs. Furthermore, Sawyer and Allison [41] mention having observed one case of DM of the “African” type in the upper Amazon region of the northeastern Peru.

The assumption that these DMs must be of African origin is not warranted, and the custom could have had both a longer history and a wider spread in the region. Unfortunately, there has been no systematic research in this phenomenon in the pre-contact archaeology of the circum-Caribbean, to the south of Mesoamerica which is characterized by a different technique of DMs [42, 43].

## Conclusions

Although DM of the “African” type in the Caribbean, as exemplified by the E-105 and likely six other individuals from Canímar Abajo, is clearly different from the Mesoamerican type, and predates any direct African influences, our lack of knowledge of its origin and dispersal requires more research before any plausible explanation can be put forward. The current findings can be tentatively interpreted in two ways:

1. As a consequence of Isthmo-Colombian influence on the pre-colonial cultures of the Greater Antilles, that has been examined on both archaeological and linguistic levels. Circulation of some plant and animal species points to an early direct communication between Lower Central America and the Greater Antilles: the presence of *pollo* maize, a variety absent in northeastern South America and Mexico, but present in Colombia and Lower Central America, has been documented in Puerto Rico [44], while a special toolkit used for processing of *Zamia* (one of the most poisonous edible plants, requiring very specific detoxification processes) has not been identified in either northeastern South America or Mexico, but only in the Isthmo-Colombian area and the Greater Antilles [45]. An examination of place names in Western Cuba based on analysis of their phonological characteristics and their comparison with Chibchan language family, which has dominated the Isthmo-Columbian region for millennia [46, 47], indicates the possibility of a Chibchan origin of the early Cuban population. The presence of the “African” type of DM as late as 1942 among Chibchan speakers in Panama [39] would be consistent with this interpretation, although it cannot be used as conclusive evidence.
2. It is also possible that chipping and filing of the incisors and canines into points developed independently within the Greater Antilles as a specific feature of insular cultures that was not brought from the continent.

In order to understand the origin and spread of chipping and filing style of DMs in the pre-contact Caribbean, individuals from archaeological sites need to be dated and properly contextualized, and a more systematic study of patterns of DMs in the region needs to be conducted.

The individual E-105 from the site of Canímar Abajo, radiometrically dated to 990–800 BC, demonstrates that the chipping and filing of anterior dentition was practiced by indigenous populations of the insular Caribbean in pre-contact times. Six other individuals dated to AD 600–800 demonstrate a similar pattern of alteration. This finding is contrary to the current assumption that this type of DM was associated exclusively with Africans brought into the Caribbean through slave trade, or their direct descendants. In each individual case of DM in the Caribbean, African origin can no longer be assumed, but needs to be demonstrated by other lines of evidence.

## Supporting Information

S1 Fig Maxillary dentition of the individual E-105. Maxillary dentition of E-105: occlusal view of the right segment of the maxilla with the central incisor of the left segment: note the reduction of the maxillary central incisors into an inverted “V” shape (photo taken at the University of Havana by MR).
(TIF)

S2 Fig Mandibular dentition of the individual E-105. Mandibular dentition of E-105, occlusal view: note the minimal degree of dental wear and alveolus indicating eruption and loss of the right M3 (photo taken at the University of Havana by MR).
(TIF)

S3 Fig Maxillary and mandibular dentition of the individual E-105 in occlusion. Composite image showing the maxillary and mandibular dentition of E-105 in occlusion, note the lack of corresponding wear on the lower anterior dentition.
(TIF)

S4 Fig Frontal view of the individual E-92. Frontal view of the lower face of the individual E-92: note the reduction of the maxillary central incisors into an inverted “V” shape. (Photo by Y. Chinique de Armas taken at the University of Havana).
(TIF)

S5 Fig Frontal view of the individual E-71. Frontal view of the lower face of the individual E-71: note the reduction of the maxillary central incisors into an inverted “V” shape and the presence of a large diastema between the maxillary central incisors. (Photo by Y. Chinique de Armas taken at the University of Havana).
(TIF)

S6 Fig Labial surface of the occlusal edge of maxillary central incisors of the individual E-105. Detail of the labial surface of the occlusal edge of maxillary central incisors of E-105: a) under 20x magnification captured by digital microscope; b) composite image captured by an SEM microscope at 80x magnification.
(TIF)

S7 Fig Lingual surface of the maxillary central incisors of the individual E-105. Lingual surface of the maxillary central incisors of E-105: a) under 20x magnification captured by digital microscope; b) composite image captured by an SEM microscope at 40x magnification. Note the smoothing and abrasion on the mesial portion of the crown spreading distally towards CEJ.
(TIF)

S8 Fig Modified dentition of individuals E-19, E-92, and E-125. Composite picture of other dentition with recorded reduction under 20x magnification (Keyance digital microscope) a) labial and b) lingual surface of the right central incisor of the individual E-19; c) labial surface of the right and d) lingual surface of the left central incisor of the individual E-92; and labial surface of the e) left and f) right central incisor of the individuals E-125.
(TIF)

S1 File Fig 2 copyright permission. (DOCX)

## References

[1] Stewart TD. Negro skeletal remains from Indian sites in the West Indies. Man. 1939;39: 49–51.

[2] Ortner DJ. A recent occurrence of an African type tooth mutilation in Florida. Am J Phys Anthropol. 1966;25(2): 177–180. 4381969 10.1002/ajpa.1330250212

[3] Stewart TD, Groome JR. The African custom of tooth mutilation in America. Am J Phys Anthropol. 1968;28: 31–42. 4297342 10.1002/ajpa.1330280112

[4] Handler JS, Corruccini RS, Mutaw RJ. Tooth mutilation in the Caribbean: Evidence from Archaeological population in Barbados. J Hum Evol. 1982;11: 297–313.

[5] Crespo E, Giusti JB. Primera evidencia de mutilación dentaria una población negroide de Puerto Rico. Revista Salud y Cultura. 1992;4(1): 95–105.

[6] Handler JS. Determining African birth from skeletal remains: A note on tooth mutilation. Historical Archaeology. 1994;28(3): 113–119.

[7] Tiesler V. New cases of an African tooth decoration from colonial Campeche, Mexico. Homo. 2002;52(3): 277–282. 12018122 10.1078/0018-442x-00034

[8] Tiesler V. La práctica Africana de la mutilación dental en las Américas. Evidencias coloniales en una población Negroide en Campeche, México. Estud Antropol Biol. 2003;11: 951–965.

[9] Price TD, Tiesler V, Burton JH. Early African diaspora in colonial Campeche, Mexico: Strontium isotope evidence. Am J Phys Anthropol. 2006;130(4): 485–490. 16444728 10.1002/ajpa.20390

[10] Schroeder H, Farmer K, Hedges REM. Reconstructing forgotten life histories: Using isotopic analysis in the study of the African Diaspora. SAfA Proceedings. 2006.

[11] Schroeder H, Haviser JB, Price TD. The Zoutsteeg three: Three new cases of African types of dental modification from Sint Maarten, Dutch Caribbean. Int J Osteoarchaeol. 2014;24(6): 688–696.

[12] Schroeder H, Ávila-Arcos MC, Malaspinas AS, David Poznik GD, Sandoval-Velasco M, Carpenter ML, et al Genome-wide ancestry of 17th-century enslaved Africans from the Caribbean. PNAS. 2015; 3669–3673. 10.1073/pnas.1421784112 25755263 PMC4378422

[13] Valcárcel Rojas R, Weston DA, Mickleburgh HL, Laffoon JE, van Duijvenbode A. El Chorro de Maita: A diverse approach to the context of diversity In: Hofman CL, van Duijvenbode A, editors. Communities in contact: Essays in archaeology, ethnohistory, and ethnography of Amerindian Circum-Caribbean. Leiden: Sidestone Press; 2011 pp. 225–251.

[14] Goose DH. Tooth-mutilation in West-Africans. Man. 1963;63: 91–93.

[15] Singer R. Artificial deformation of teeth: A preliminary report. S Afr J Sci. 1953;50: 116–122.

[16] Van Reenen JF. Tooth Mutilating and Extraction Practices Amongst the Peoples of South West Africa (Namibia) In: Singer R, Lundy JK, editors. Variation, Culture and Evolution in African Populations. Johannesburg: Witwatersrand University Press; 1986 pp. 159–169.

[17] Wasterlain SN, Neves MJ and Ferreira MT. Dental Modifications in a Skeletal Sample of Enslaved Africans Found at Lagos (Portugal). Int J Osteoarchaeol. 2015; 10.1002/oa.2453

[18] Roksandic M, Buhay WM, Chinique des Armas Y, Rodríguez Suárez R, Peros MC, Roksandic I, et al Radiocarbon and stratigraphic chronology at Canímar Abajo, Matanzas, Cuba. Radiocarbon. 2015;57(5): 1–9.

[19] Klein HS. The Atlantic slave trade. 2nd edition Cambridge: Cambridge University Press; 2010.

[20] Milner GR, Larsen CS. Teeth as artifacts of human behavior In: Kelley MA Larsen SC, editors. Advances in Dental Anthropology. New York: Wiley-Liss; 1991 pp. 357–378.

[21] Roksandic M. Position of skeletal remains as a key to understanding mortuary behavior In: Haglund WD, Sorg MH, editors. Advances in forensic taphonomy: Method, theory, and archaeological perspectives. Boca Raton: CRC Press; 2002 pp. 99–117.

[22] Roksandic M, Armstrong SD. Using the life history model to set the stage(s) of growth and senescence in bioarchaeology and paleodemography. Am J Phys Anthropol. 2011;145(3): 337–347. 10.1002/ajpa.21508 21469078

[23] Buikstra JE, Ubelaker DH. Standards for data collection from human skeletal remains. Research Series, no.44. Fayetteville: Arkansas Archaeological Survey; 1994.

[24] Bonfiglioli B, Mariotti V, Facchini F, Belcastro MG, Condemi S. Masticatory and non-masticatory dental modifications in the epipalaeolithic necropolis of Taforalt (Morocco). Int J Osteoarchaeology. 2004;14: 448–546.

[25] Molnar P. Extramasticatory dental wear reflecting habitual behavior and health in past populations. Clin Oral Investig. 2011;15: 681–689. 10.1007/s00784-010-0447-1 20706752

[26] Alt KW, Pichler SL. Artificial modifications of human teeth In: Alt KW, Rosen FW, Teschler-Nicola M, editors. Dental anthropology, fundamentals, limits, and prospects. New York: Springer-Wein; 1998 pp. 387–415.

[27] Lee JJW, Constantino PJ, Lucas PW, Lawn BR. Fracture in teeth—a diagnostic for inferring bite force and tooth function. Biological Reviews. 2011;86(4): 959–974. 10.1111/j.1469-185X.2011.00181.x 21507194

[28] Scott GR, Winn JR. Dental chipping: Contrasting patterns of microtrauma in Inuit and European populations. Int J Osteoarchaeol. 2011; 21(6): 723–731.

[29] Constantino PJ, Lee JJW, Chai H, Zipfel B, Ziscovici C, Lawn BR, Lucas PW. Tooth chipping can reveal the diet and bite forces of fossil hominins. Biol Lett 2010;6: 826–829. 10.1098/rsbl.2010.0304 20519197 PMC3001363

[30] Andrefsky W. Lithics: Macroscopic approaches to analysis. 2nd edition Cambridge University Press: Cambridge; 2005.

[31] Havill LM, Warren DM, Jacobi KP, Gettelman KD, Cook DC, Pyburn KA. Late Classic tooth filing at Chau Hiix and Tipu, Belize In: Whittington SL, Reed DM, editors. Bones of the Maya: Studies of ancient skeletons. Tuscaloosa: University of Alabama Press; 1997 pp. 89–104.

[32] Herrmann NP, Benedix DC, Scott AM, and Haskins VA. A brief comment on functional use of intentionally modified tooth from the Rio Talgua region, Northeastern Honduras. Dental Anthropology. 1999;13(2): 9–12.

[33] Turner CG, Machado LMC. A new dental wear pattern and evidence for high carbohydrate consumption in a Brazilian archaic skeletal population. Am J Phys Anthropol. 1983;61: 125–130. 6869509 10.1002/ajpa.1330610113

[34] Irish JD, Turner CG. More lingual surface attrition of the maxillary anterior teeth in American Indians: Prehistoric Panamanians. Am J Phys Anthropol. 1987;73(2): 209–213. 3303956 10.1002/ajpa.1330730207

[35] Saul JM, Saul FP. Preclassic skeletons from Cuello In: Whittington SL, Reed DM, editors. Bones of the Maya: Studies of Ancient Skeletons. Tuscaloosa: University of Alabama Press; 1997 pp. 28–50.

[36] Irish JD, Turner CG. Brief communication: first evidence of LSAMAT in non-native Americans: historic Senegalese from West Africa. Am J Phys Anthropol. 1997;102(1): 141–146. 9034045 10.1002/(SICI)1096-8644(199701)102:1<141::AID-AJPA12>3.0.CO;2-0

[37] Chinique de Armas Y, Buhay WM, Rodríguez Suárez R, Bestel S, Smith D, Mowat SD, Roksandic M. Starch analysis and isotopic evidence of consumption of cultigens among fisher-gatherers in Cuba: the archaeological site of Canímar Abajo, Matanzas. J Archaeol Sci. 2015;58: 121–132.

[38] Buxton LHD, Trevor JC, Julien AH. Skeletal remains from the Virgin Islands. Man. 1938;38: 49–52.

[39] Stewart TD. Persistence of the African type of tooth pointing in Panama. Am Anthropol. 1942;44(2): 328–330.

[40] Farabee WC. The Arawaks of Northern Brazil and Southern British Guiana. Am J Phys Anthropol. 1918;1(4): 427–442.

[41] Sawyer DR, Allison MJ. Tooth mutilation in pre-Columbian Peru and Chile and Modern-day Nigeria. Ann Dent. 1992;51(1): 24–26. 1352958

[42] Rubín de la Borbolla DF. Types of tooth mutilation found in Mexico. Am J Phys Anthropol. 1940;26: 349–365.

[43] Romero J. Dental mutilation, trephination, and cranial deformation In: Stewart TD, editor. Handbook of middle American Indians. Vol. 9 Austin: University of Texas Press; 1970 pp. 50–69.

[44] Pagán Jiménez JR, Rodríguez Ramos R, Reid BA, van den Bel M, Hofman CL. Early dispersals of maize and other food plants into the Southern Caribbean and Northeastern South America. Quat Sci Rev. 2015;123: 231–246.

[45] Rodríguez Ramos R. Isthmo-Antillean engagements In: Keegan WF, Hofman CL, Rodríguez Ramos R, editors. The Oxford handbook of Caribbean archaeology. Oxford: Oxford University Press; 2013 pp. 155–170.

[46] Constenla Umaña A. Chibchan languages In: Campbell L, Grondona V, editors. The indigenous languages of South America: A comprehensive guide. Berlin: Mouton; 2012 pp. 391–440.

[47] Roksandic I. Reconsideration of Taíno and Pre-Taíno Toponymy in Cuba. Proceedings of the 25th Congress of the International Association for Caribbean Archaeology. San Juan, Universidad de Puerto Rico; 2015.

