# Supplementary figures and images for: Not of African Descent: Dental Modification among Indigenous Caribbean People from Canímar Abajo, Cuba
Source: PLoS One. 2016 Apr 12;11(4):e0153536. doi: 10.1371/journal.pone.0153536 (PMC4829177; doi:10.1371/journal.pone.0153536)

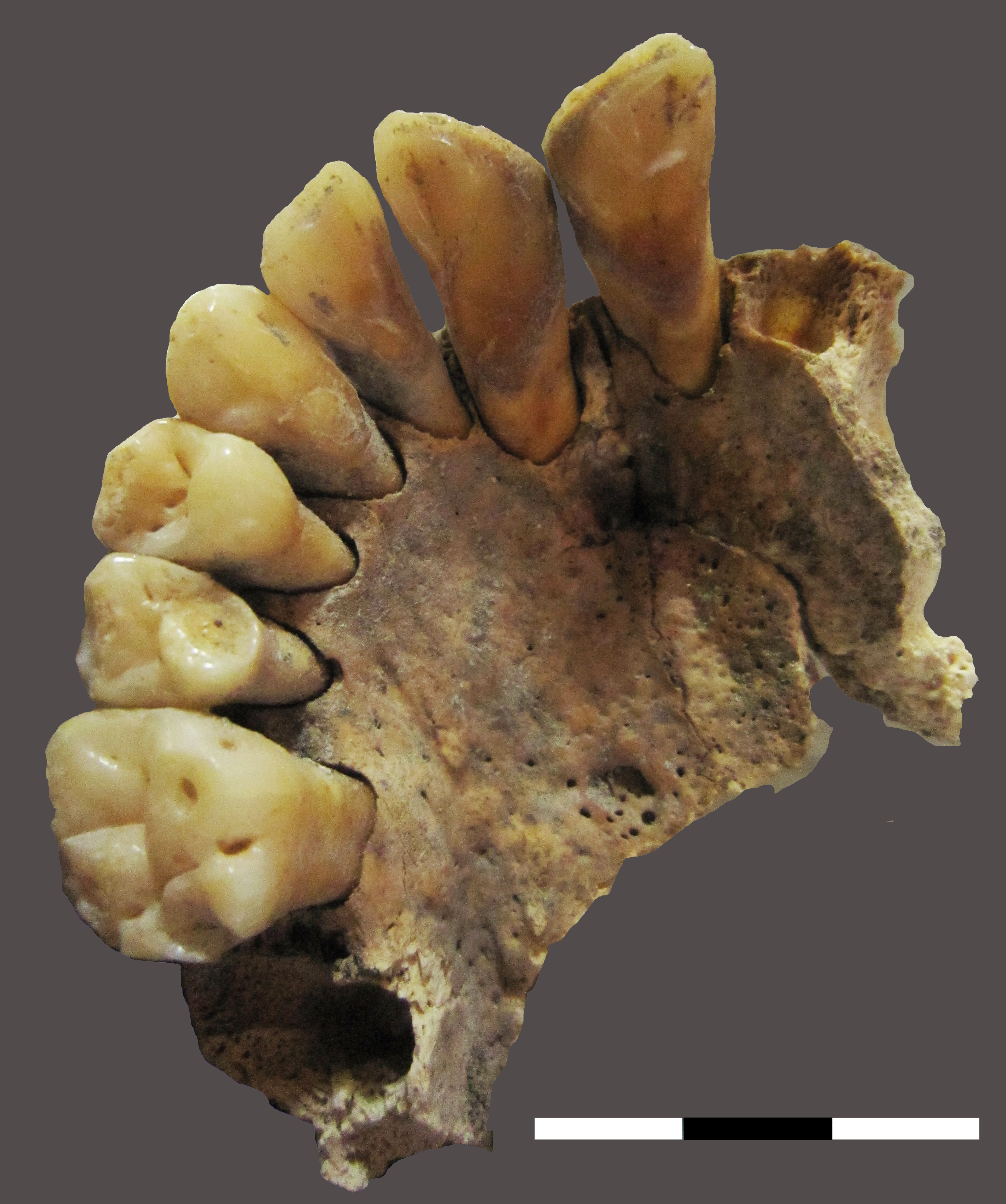

Supplement: S1 Fig — Maxillary dentition of E-105: occlusal view of the right segment of the maxilla with the central incisor of the left segment: note the reduction of the maxillary central incisors into an inverted “V” shape (photo taken at the University of Havana by MR). (TIF) [file pone.0153536.s001.tif]

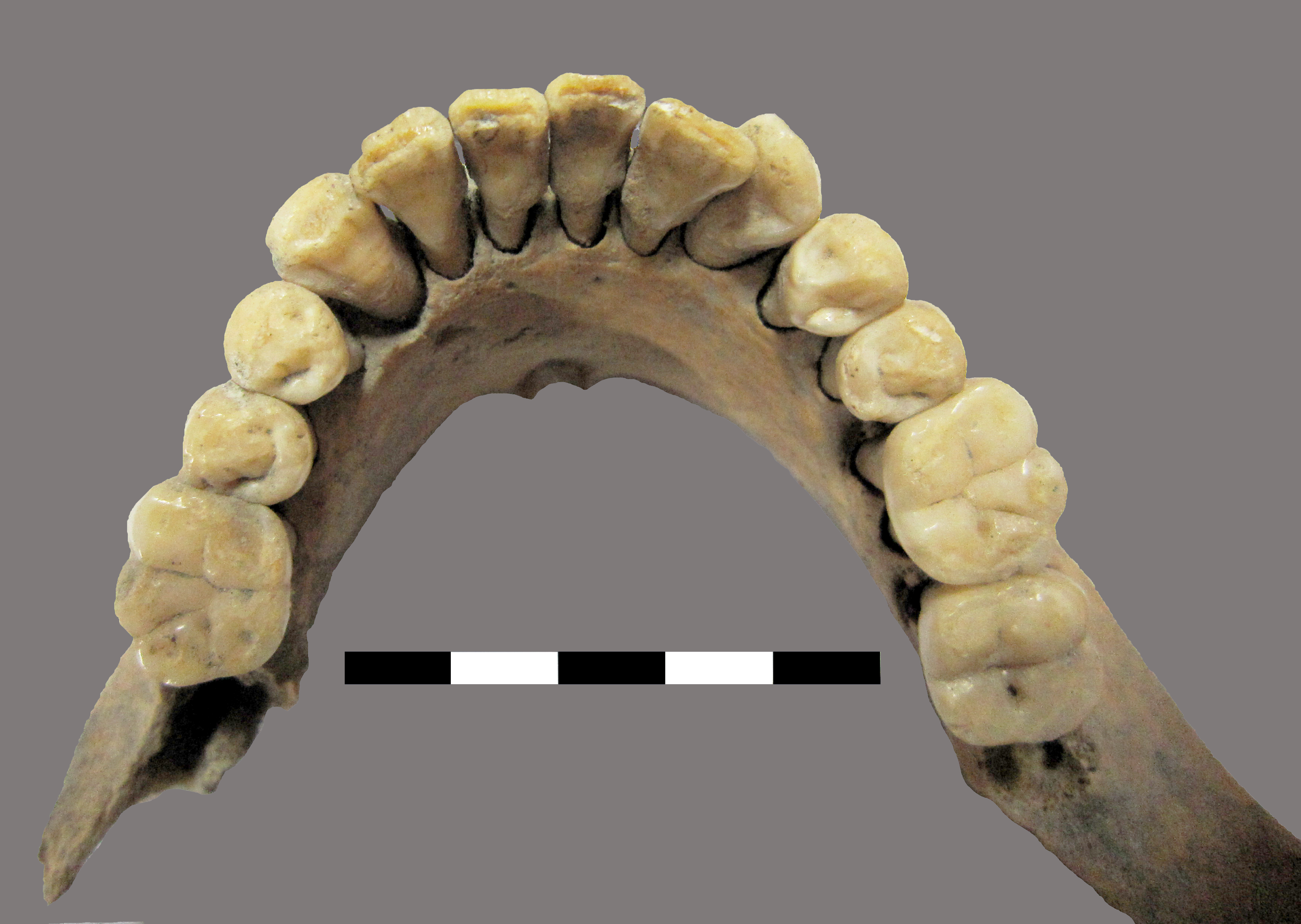

Supplement: S2 Fig — Mandibular dentition of E-105, occlusal view: note the minimal degree of dental wear and alveolus indicating eruption and loss of the right M3 (photo taken at the University of Havana by MR). (TIF) [file pone.0153536.s002.tif]

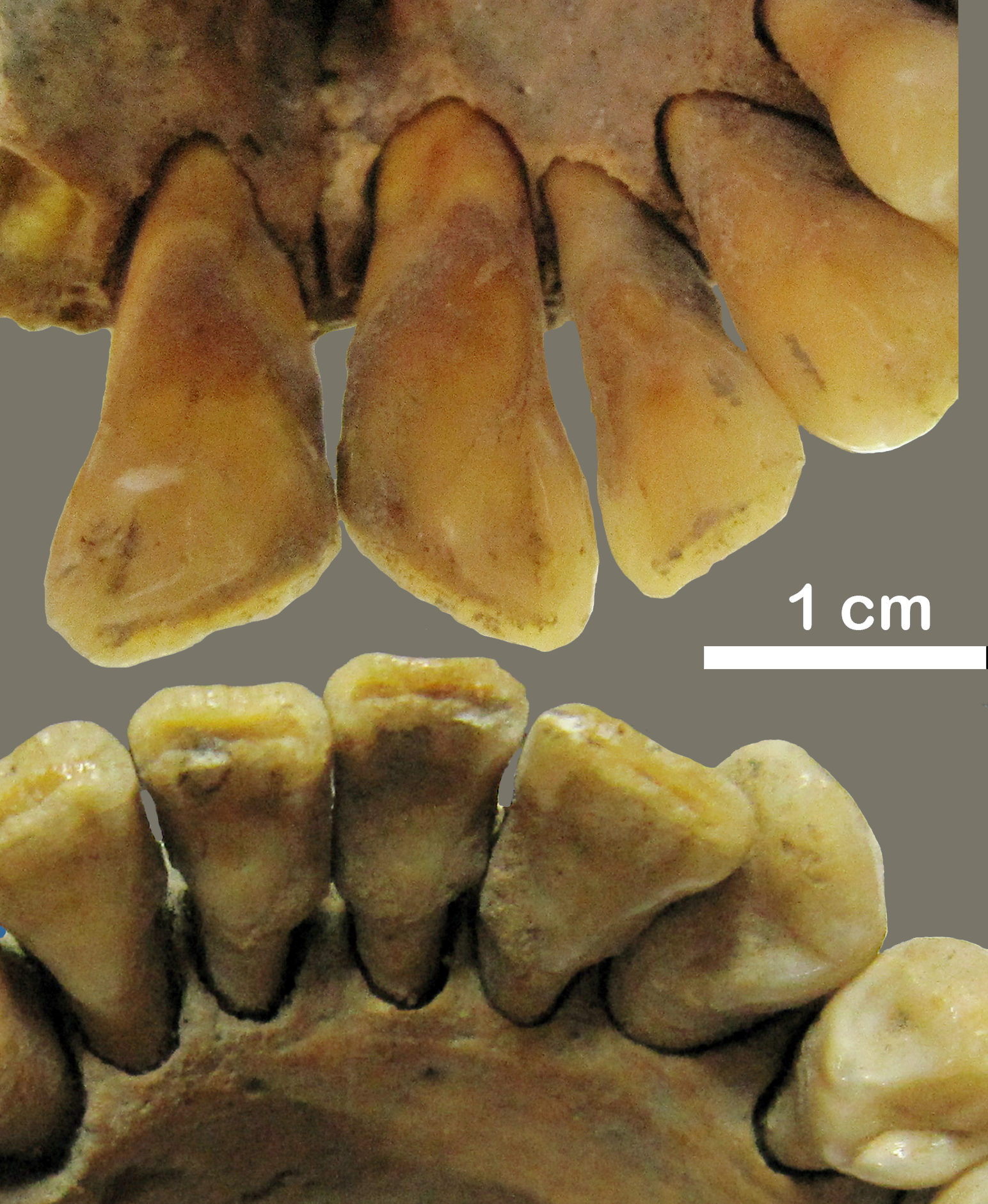

Supplement: S3 Fig — Composite image showing the maxillary and mandibular dentition of E-105 in occlusion, note the lack of corresponding wear on the lower anterior dentition. (TIF) [file pone.0153536.s003.tif]

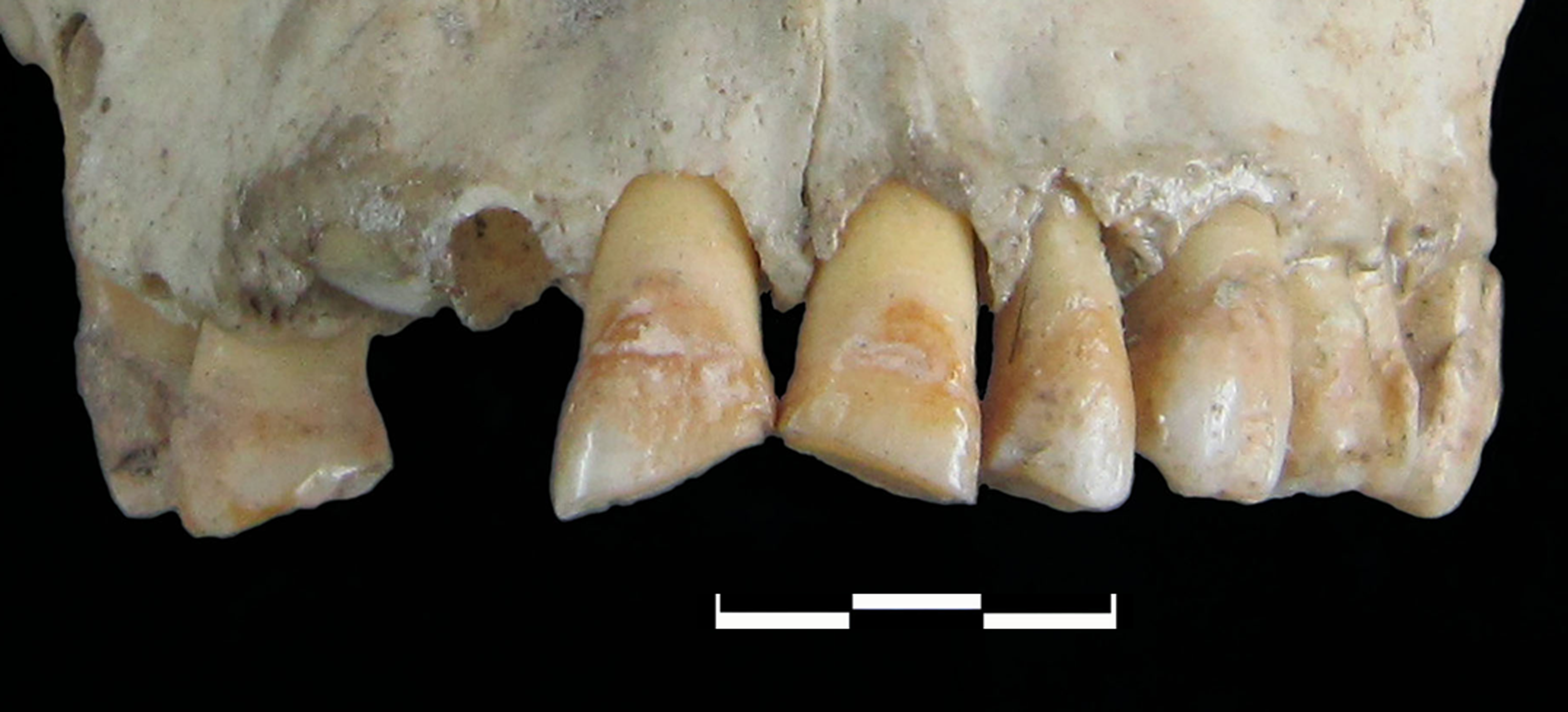

Supplement: S4 Fig — Frontal view of the lower face of the individual E-92: note the reduction of the maxillary central incisors into an inverted “V” shape. (Photo by Y. Chinique de Armas taken at the University of Havana). (TIF) [file pone.0153536.s004.tif]

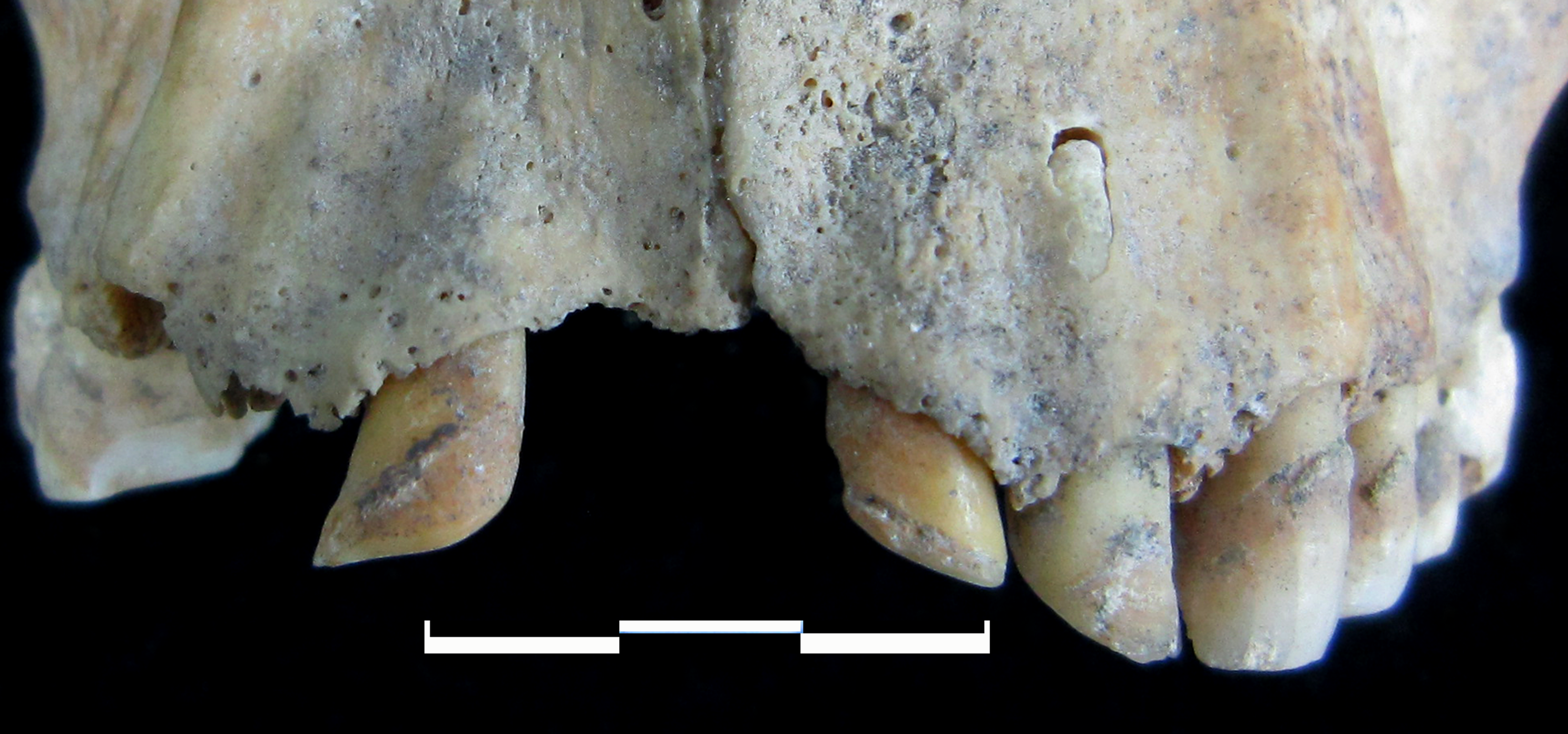

Supplement: S5 Fig — Frontal view of the lower face of the individual E-71: note the reduction of the maxillary central incisors into an inverted “V” shape and the presence of a large diastema between the maxillary central incisors. (Photo by Y. Chinique de Armas taken at the University of Havana). (TIF) [file pone.0153536.s005.tif]

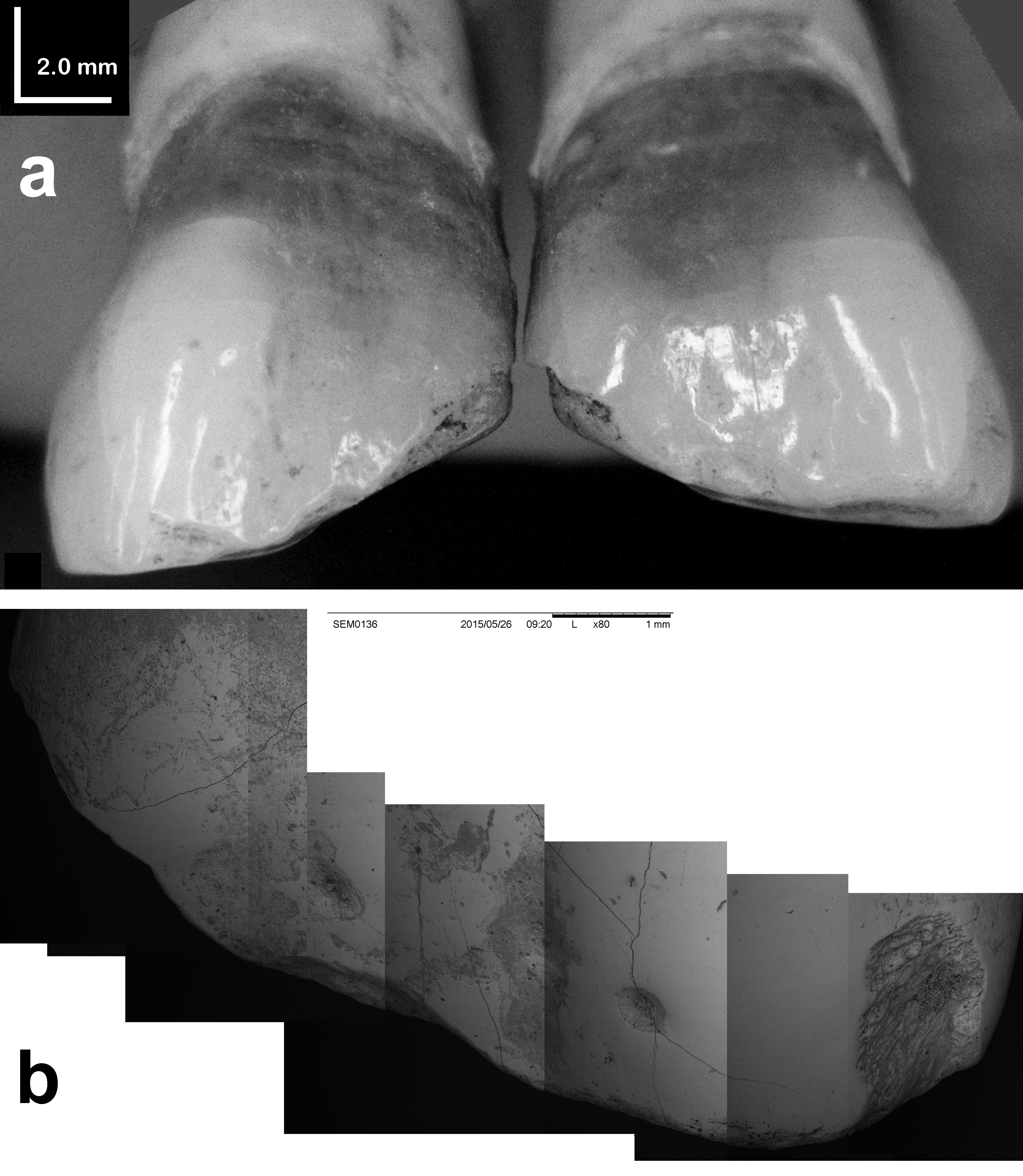

Supplement: S6 Fig — Detail of the labial surface of the occlusal edge of maxillary central incisors of E-105: a) under 20x magnification captured by digital microscope; b) composite image captured by an SEM microscope at 80x magnification. (TIF) [file pone.0153536.s006.tif]

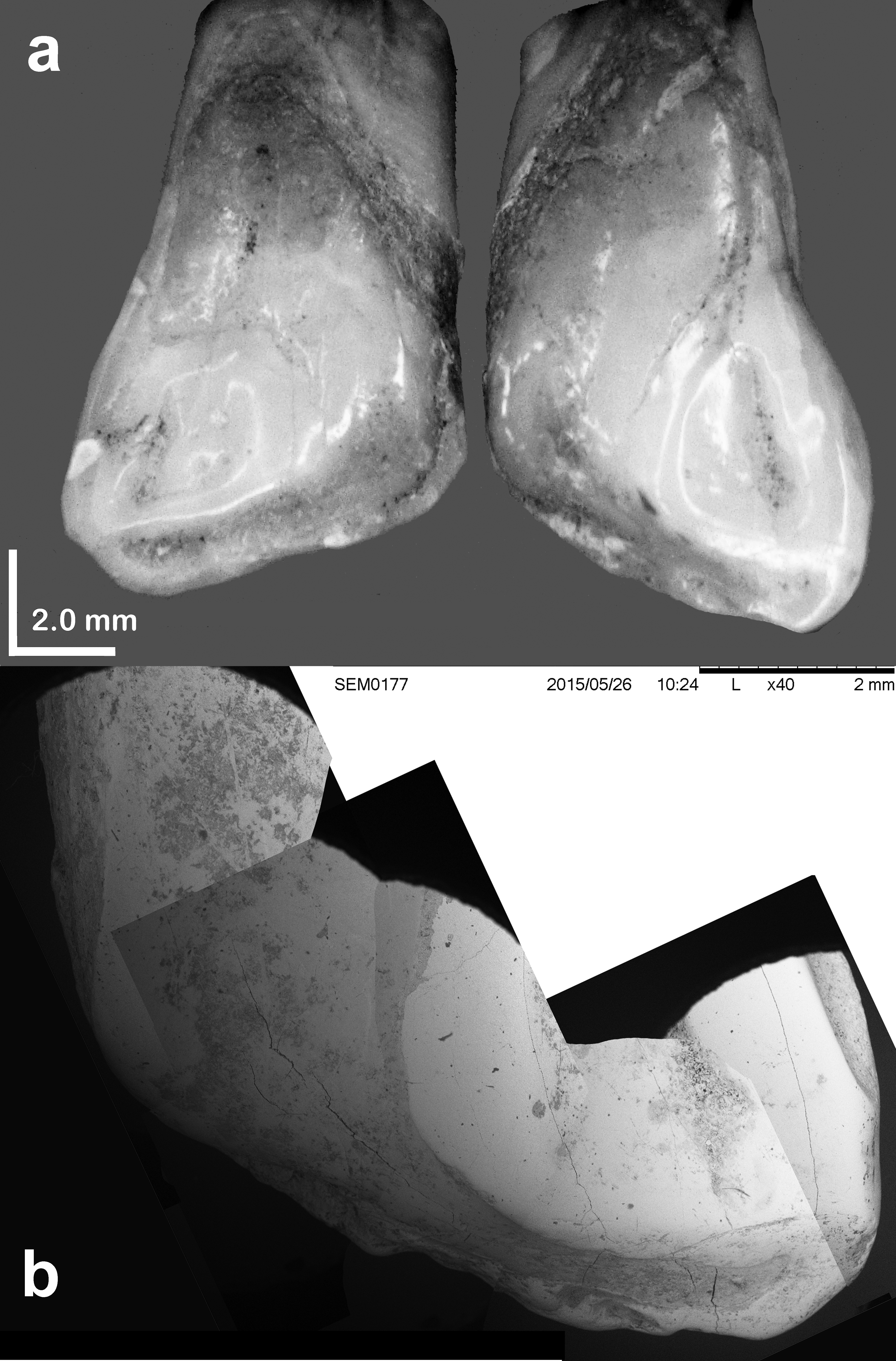

Supplement: S7 Fig — Lingual surface of the maxillary central incisors of E-105: a) under 20x magnification captured by digital microscope; b) composite image captured by an SEM microscope at 40x magnification. Note the smoothing and abrasion on the mesial portion of the crown spreading distally towards CEJ. (TIF) [file pone.0153536.s007.tif]

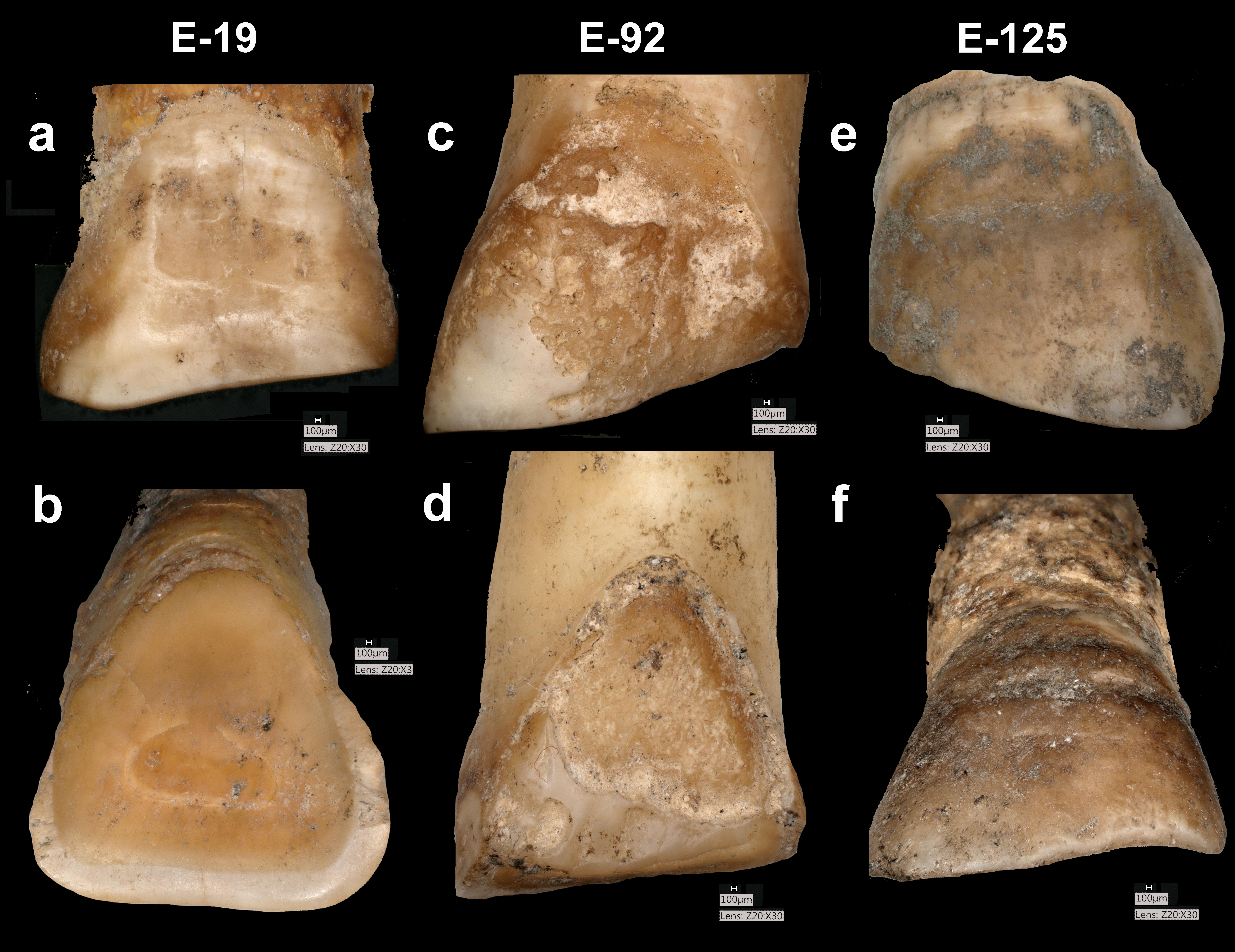

Supplement: S8 Fig — Composite picture of other dentition with recorded reduction under 20x magnification (Keyance digital microscope) a) labial and b) lingual surface of the right central incisor of the individual E-19; c) labial surface of the right and d) lingual surface of the left central incisor of the individual E-92; and labial surface of the e) left and f) right central incisor of the individuals E-125. (TIF) [file pone.0153536.s008.tif]
