# Supplementary material for: Not of African Descent: Dental Modification among Indigenous Caribbean People from Canímar Abajo, Cuba
Source: PLoS One. 2016 Apr 12;11(4):e0153536. doi: 10.1371/journal.pone.0153536 (PMC4829177; doi:10.1371/journal.pone.0153536)
Supplement: S1 File — (DOCX) [file pone.0153536.s009.docx]

| \| **Mcclure, Mark E - (mcclurem)** \| \| --- \| |  |
| --- | --- | --- |
|  |  |
| \| \| to me   \| \| --- \| \| \| --- \| --- \|   Dear Mirjana,  Yes, you have permission to use the Figure you cite.  Best,  Mark    Mark McClure RADIOCARBON Editorial Manager Univ. of Arizona, Dept. of Geosciences 4717 E. Fort Lowell Rd, #104 Tucson, AZ  USA 85712 [+1 (520) 621-0641](tel:%2B1%20%28520%29%20621-0641) phone \| [+1 (520) 621-0584](tel:%2B1%20%28520%29%20621-0584) fax [www.radiocarbon.org](http://www.radiocarbon.org/) \| <https://journals.uair.arizona.edu/index.php/radiocarbon/index>  Follow us on Twitter: <https://twitter.com/14Cjournal>  On Feb 24, 2016, at 6:13 PM, Mirjana Roksandic <[mroksand@gmail.com](mailto:mroksand@gmail.com)> wrote:  Dear Mark,  Here is the formal request:  I request permission for the open-access journal PLOS ONE to publish *the adjusted figure 1 from Roksandic et al. 2015 published in Radiocarbon 57(5) on page 575* under the Creative Commons Attribution License (CCAL) CC BY 4.0 (<http://creativecommons.org/licenses/by/4.0/>). Please be aware that this license allows unrestricted use and distribution, even commercially, by third parties. Please reply and provide explicit written permission to publish *adjusted figure 1 from Roksandic et al. 2015 published in Radiocarbon 57(5) on page 575* under a CC BY license.”  Please confirm and I will included it with my paper  Thank you for your understanding  m. | |
